# Evaluation of the impact of cardiopulmonary rehabilitation exercise training on cardiopulmonary function in patients with chronic obstructive pulmonary disease complicated by unstable angina pectoris using a hierarchical deep learning CT image model

**DOI:** 10.3389/fphys.2026.1735687

**Published:** 2026-02-18

**Authors:** Kongyu Xing, Chunmiao Tan, Xiaoling Cheng, Fen Jiang

**Affiliations:** 1 The First Affiliated Hospital of Hainan Medical University, Haikou, Hainan, China; 2 Quanzhou Third Hospital, Quanzhou, China; 3 School of Nursing, Hainan Medical University, Haikou, Hainan, China

**Keywords:** COPD, CPR exercise training, CT, deeplearning, unstable angina pectoris

## Abstract

**Objective:**

This study aimed to quantitatively evaluate the impact of cardiopulmonary rehabilitation (CPR) exercise training on cardiopulmonary structure and function in patients with chronic obstructive pulmonary disease (COPD) complicated by unstable angina (UA) pectoris, based on a hierarchical deep learning (DL) CT image model.

**Methods:**

This prospective randomized controlled trial enrolled 400 patients with COPD complicated by UA pectoris, stratified according to GOLD grades (I-IV), who were randomly allocated to an experimental group (EG, n = 200, receiving 12 weeks of standard CPR training) and a control group (CG, n = 200, receiving conventional care). A multi-task 3D U-Net + ResNet50 DL model was constructed to automatically quantify four categories of imaging biomarkers from chest high-resolution CT (HRCT) and coronary CT angiography images: lung parenchyma (percentage of low attenuation volume, LAV%, 15th percentile density Perc15, mean lung density, MLD), airways (percentage of airway wall thickness WA%, Pi10), pulmonary vasculature (percentage of blood vessels <5 mm in cross-sectional area BV%, vascular fractal dimension), and heart (coronary artery calcium score, CACS, left ventricular mass, LVM, ejection fraction, EF, stroke volume, SV). Concurrently, pulmonary function, cardiopulmonary exercise testing parameters, and 6-min walk distance (6MWD) were assessed at baseline, 6 weeks, and 12 weeks of the intervention.

**Results:**

Versus CG, after 12 weeks of intervention, EG demonstrated notable imaging improvements across all GOLD grades: decreased LAV%, increased Perc15 and MLD; reduced WA% and Pi10; increased BV5% and vascular fractal dimension; improved EF and SV, and decreased LVM (all *P* < 0.05). Clinically, EG also showed substantially better FEV_1_, FVC, peak VO_2_, and 6MWD than CG (*P* < 0.01). Correlation analysis revealed moderate to strong correlations between these imaging metrics and clinical functional parameters (|*r*| = 0.36–0.62, *P* < 0.001). The constructed DL model demonstrated excellent segmentation accuracy (Dice coefficient: 0.87–0.95) and quantification reliability in both internal and external validations.

**Conclusion:**

CPR not only substantially improves clinical cardiopulmonary function but also induces beneficial structural remodeling of the lung parenchyma, airways, pulmonary vasculature, and heart in patients with COPD complicated by UA pectoris. The DL-based CT image quantification framework provides reliable imaging biomarkers for objectively evaluating rehabilitation efficacy in these comorbid patients, offering new evidence for personalized management of cardiopulmonary comorbidities.

## 1 Introduction

Chronic obstructive pulmonary disease (COPD) is a chronic respiratory disease characterized by persistent airflow limitation, primarily caused by airway and lung inflammation in response to harmful particles or gases (Yildirim et al., 2025). The global initiative for chronic obstructive lung disease (GOLD) emphasizes that COPD, with its high morbidity and disability rates, is a leading cause of death (Agustí et al., 2023). Furthermore, according to the GOLD grading criteria, as COPD progresses from GOLD stage 1 to stage 4, patients experience a gradual decline in pulmonary ventilation function and exercise tolerance, alongside a substantially increased risk of cardiovascular complications (Silva et al., 2024; Li T. et al., 2025).

Among cardiovascular diseases, unstable angina (UA) has a higher incidence tendency in patients with COPD (de Miguel-Díez et al., 2024). Their shared pathophysiological basis includes systemic inflammatory response, oxidative stress, increased sympathetic nerve activity, and endothelial dysfunction induced by hypoxia (Polman et al., 2024). This cardiopulmonary comorbidity not only exacerbates the degree of myocardial ischemia but also substantially reduces cardiopulmonary reserve capacity during acute episodes, adversely affecting prognosis (Hawkins et al., 2024). Therefore, the comprehensive management of COPD complicated by UA must simultaneously address the assessment and intervention of both respiratory and cardiovascular function.

Pulmonary rehabilitation and cardiac rehabilitation have been demonstrated to substantially improve exercise tolerance, symptoms, and quality of life in patients with chronic respiratory and cardiovascular diseases (Jenkins et al., 2024; Hua et al., 2024). For patients with COPD complicated by UA, coordinated cardiopulmonary rehabilitation (CPR) is expected to yield synergistic benefits by improving muscle metabolism, reducing ventilatory load, and optimizing cardiac hemodynamics. However, existing research predominantly focuses on individual systems, and high-quality evidence using objective imaging endpoints for combined rehabilitation interventions in this complex comorbid population remains scarce (Al-Ajlouni et al., 2024). Furthermore, while traditional functional assessments are valuable, their sensitivity to key pathological changes such as lung parenchymal and cardiac structural/perfusion alterations is limited, making it difficult to comprehensively reveal the interconnected changes in cardiopulmonary structure and function (Dal Negro et al., 2025).

In recent years, cardiothoracic high-resolution CT (HRCT) has enabled the simultaneous acquisition of information on lung parenchyma (emphysema, airways), pulmonary vasculature, coronary artery calcification, and cardiac chamber/myocardial structure, providing an important tool for studying cardiopulmonary disease interactions (Rathore et al., 2025; Sui et al., 2025). Concurrently, deep learning (DL) has seen rapid progress in medical image processing (segmentation, quantification, risk prediction) and is widely applied in the automatic quantification of pulmonary nodules, emphysema distribution, airway metrics, coronary artery lesions, and ventricular geometry (Tsampras et al., 2025). While DL for segmentation and characterization in cardiothoracic CT has matured substantially, its application in evaluating rehabilitation efficacy in patients with COPD (stratified by GOLD grade) complicated by UA remains an emerging field. Key challenges include standardizing multi-phase/multi-modal data, modeling the relationship between cardiopulmonary imaging metrics and clinical outcomes, and ensuring model generalizability and interpretability across populations with varying GOLD grades and coronary artery disease burden. Recent approaches aim to enhance clinical utility through multi-center validation, lightweight networks, and interpretability modules, and attempt to integrate imaging metrics with clinical endpoints for efficacy evaluation and subgroup analysis (De Santis et al., 2025).

Based on this, this study established a DL-driven quantitative cardiothoracic CT assessment system to evaluate the impact of systematic CPR training on cardiopulmonary function in patients with COPD (stratified by GOLD grade) complicated by UA, using objective imaging endpoints. The study will enroll participants stratified by GOLD grade, automatically extract lung parenchyma, airway, pulmonary vasculature, coronary artery, and ventricular structural metrics, and compare the correlations between imaging parameters and clinical cardiopulmonary function before and after the intervention, while assessing response differences across subgroups with varying GOLD grades and coronary artery disease burden. This aims to provide quantifiable biomarkers for personalized exercise prescription, efficacy monitoring, and the management of high-risk patients with cardiopulmonary comorbidities.

## 2 Research methodologies

### 2.1 Research design

This prospective, single-center randomized controlled trial aimed to evaluate the impact of CPR training on cardiopulmonary function in patients with COPD complicated by UA pectoris, while performing quantitative analysis of chest/cardiac CT images via a DL model. The study strictly adhered to the ethical principles of the Declaration of Helsinki and was approved by the local institutional ethics committee. Written informed consent was obtained from all participants prior to enrollment.

### 2.2 Research object

This study prospectively enrolled patients from the CPR Center and the joint cardiology-pulmonology outpatient clinic of The First Affiliated Hospital of Hainan Medical University between April 2021 and April 2024. Inclusion criteria were: age ≥40 years; diagnosis of COPD meeting the GOLD 2023 criteria and complicated by UA; confirmation by chest HRCT and coronary CT angiography (CTA); ability to complete CPR training; and willingness to undergo HRCT/CTA examinations and provide written informed consent. Exclusion criteria included: severe heart failure (meeting any of the following criteria: *New York Heart Association* [NYHA] class III–IV; or left ventricular ejection fraction [LVEF] ≤ 35% measured by echocardiography within the past 6 months; or clinical evidence of decompensated heart failure requiring hospitalization or intravenous diuretics within the previous 3 months [e.g., dyspnea at rest, orthopnea, peripheral edema]), valvular heart disease, lung cancer, or recent major surgery; myocardial infarction or severe unstable angina within the preceding 6 months; presence of unstable arrhythmias (such as symptomatic ventricular tachycardia, high-grade atrioventricular block) or other life-threatening cardiac conditions (including uncontrolled severe heart failure [NYHA class IV], hypertrophic obstructive cardiomyopathy, acute myocarditis, severe aortic stenosis, cardiac tamponade, or malignant arrhythmias, etc.); pneumonia or incomplete recovery from a COPD exacerbation within the last 8 weeks; comorbid severe diseases (e.g., malignant tumors, end-stage renal disease) with an expected survival of <5 years; pregnancy, lactation, or planned pregnancy; and inability to comply with the study protocol; pneumonia or incomplete recovery from a COPD exacerbation within the last 8 weeks; comorbid severe diseases (e.g., malignant tumors, end-stage renal disease) with an expected survival of <5 years; pregnancy, lactation, or planned pregnancy; and inability to comply with the study protocol.

### 2.3 Sample size calculation

The change in VO_2_max before and after the intervention served as the primary reference outcome in this study. Based on previous literature (2023–2025 multicenter COPD rehabilitation studies) (Bilberg et al., 2024), similar interventions have been reported to increase VO_2_max by approximately 3–4 mL kg^-1^·min^-1^ on average, with a standard deviation of about 5 mL kg^-1^·min^-1^. Assuming a between-group difference (experimental group, EG vs. control group, CG) of 3 mL kg^-1^·min^-1^, with α = 0.05 (two-tailed) and a power (1–β) of 0.8, the sample size per group calculated using the formula for the independent samples t-test was:

$$n=\frac{2\sigma^{2}{\left(Z_{1-\alpha /2}+Z_{1-\beta }\right)}^{2}}{\delta^{2}}$$ (1)

where *σ* = 5, *δ* = 3, *Z*_1_-*α*/_2_ = 1.96, and *Z*_1_-*β* = 0.84, yielding n ≈ 44 per group. To ensure sufficient statistical power across all GOLD grade subgroups (I-IV) and account for the stratified design and an estimated 15% dropout rate, the sample size was increased to 50 per subgroup, resulting in a total sample size of approximately 400 participants (200 in EG and 200 in CG). This sample size is adequate for analyzing both the primary endpoint and subgroup analyses.

### 2.4 Grouping method

Enrolled patients were stratified according to GOLD 2023 grades (I-IV). Within each stratum, a random allocation sequence was generated using R software to assign patients in a 1:1 ratio to either, EG (CPR training group) or CG (conventional treatment group). The allocation results were managed by the study coordinator, and personnel implementing the interventions were blinded to the assignment until after allocation to prevent selection bias. Ultimately, a total of 400 patients were allocated, with 200 in, EG and 200 in CG. The distribution across GOLD subgroups was as follows: GOLD I: 48 patients (24 EG, 24 CG), GOLD II: 106 patients (53 EG, 53 CG), GOLD III: 156 patients (78 EG, 78 CG), GOLD IV: 90 patients (45 EG, 45 CG). The GOLD grading criteria were: GOLD I (Mild): FEV_1_ ≥ 80% predicted; GOLD II (Moderate): 50% ≤ FEV_1_<80% predicted; GOLD III (Severe): 30% ≤ FEV_1_<50% predicted; GOLD IV (Very Severe): FEV_1_<30% predicted or presence of respiratory failure.

### 2.5 Intervention methods

CG received conventional pharmacological treatment and care, including guidance on the use of bronchodilators, inhaled corticosteroids, antibiotics, as well as routine nursing and follow-up.

EG, in addition to CG regimen, underwent a 12-week CPR program, administered three times per week for 60 min per session. The program consisted of the following four core components:

1. Aerobic exercise training included walking, cycling, and elliptical training, adjusted to moderate intensity (50%–70% of VO_2_max) based on the patient’s VO_2_max and Borg scale score, initially lasting 30–40 min per session and gradually increasing to 60 min.
2. Strength training referred to upper and lower limb machine-based exercises (e.g., leg press, seated row, chest press) at an intensity of 50%–70% of 1RM, performed for 20–30 min per session, 2–3 times per week.
3. Respiratory muscle training included diaphragmatic breathing, pursed-lip breathing, and respiratory muscle endurance training, conducted for 10–15 min per session, daily.
4. Health education and nutritional guidance covered COPD pathophysiology, medication use, inhaler technique, smoking cessation counseling, nutritional support, and psychological counseling, provided once weekly for 12 weeks.

### 2.6 Imaging acquisition and processing

#### 2.6.1 CT scanning protocol

To ensure image quality and quantitative analysis accuracy, this study utilized HRCT and coronary CTA for image acquisition:

HRCT scanning was performed using a ≥64-slice multi-detector spiral CT scanner. The tube voltage was set to 120 kV, and automatic tube current modulation was used in low-dose mode. The scan slice thickness and interval were both 1 mm, with the scan range extending from the lung apex to the diaphragm. Patients performed a full inspiratory breath-hold to ensure consistent lung volume. Images were reconstructed using a high-frequency bone reconstruction kernel to improve the resolution of small airways and lung structures.

CTA scanning was performed using a 64-slice or higher multi-detector spiral CT scanner, supporting ECG-triggered scanning to ensure clear coronary artery image acquisition during diastole. Scanning parameters were set to a tube voltage of 100–120 kV, with tube current automatically adjusted based on patient body habitus, and a slice thickness of 0.75 mm. An iodine-based contrast agent was administered at an injection rate of 4–5 mL/s, with a total dose of 70–100 mL, delivered via a dual-syringe automatic injector. After acquisition, images underwent multi-planar reconstruction, maximum intensity projection, and volume rendering processing.

#### 2.6.2 Image preprocessing

To ensure the robustness and accuracy of DL model training, all HRCT and CTA images underwent standardized preprocessing. First, images were denoised using Gaussian filtering (σ = 1–1.5), and Hounsfield Unit (HU) values were uniformly truncated to the range of [-1,000, 400], followed by linear normalization to [0, 1] for model input.

### 2.7 Construction of DL model

#### 2.7.1 Model architecture

This study constructed a multi-branch DL architecture based on 3D U-Net and ResNet50 for multi-task automated quantitative analysis of the lungs, airways, pulmonary vasculature, and heart (see structural diagram 1). The overall network employs a multi-task learning (MTL) strategy, utilizing a shared encoder to extract global and local deep features, and outputs different anatomical structures and functional metrics via independent decoder branches. This enables joint modeling of structure and function, enhances feature representation capability, and reduces the risk of overfitting. The specific design is as follows:

The lung parenchyma quantification branch, based on 3D U-Net, was designed for lung lobe and low attenuation area (LAA) segmentation, and calculated the percentage of low attenuation volume (LAV%), the 15th percentile density (Perc15), and the mean lung density (MLD). The encoder captured global lung structural information through multi-scale convolutions, while the decoder utilized skip connections to preserve spatial resolution, outputting continuous voxel probability maps for precise quantification.

The airway quantification branch utilized a ResNet50 residual network to extract local airway features, automatically calculating the percentage of airway wall thickness (WA%), Pi10, lumen diameter, and the wall area to lumen area ratio (WA/LA). Through multi-scale feature fusion and an edge enhancement module, the segmentation accuracy of small bronchi was improved. In the post-processing step, Pi10 was calculated using the WA/LA and lumen diameter data to achieve quantification of airway remodeling.

The pulmonary vasculature quantification branch employed a 3D convolutional network combined with image enhancement and vessel tracking algorithms to achieve small vessel segmentation and extraction of BV5% (percentage of blood vessels <5 mm in cross-sectional area) and vascular fractal dimension. The network leveraged multi-scale contextual information to enhance microvessel detection, while outputting continuous voxel probability maps for calculating vascular complexity metrics.

The cardiac quantification branch utilized a 3D U-Net to segment the left and right ventricles and myocardium, incorporating an attention mechanism (AM) to enhance boundary recognition accuracy. The branch outputs included the coronary artery calcium score (CACS), left ventricular mass (LVM), ejection fraction (EF), and stroke volume (SV). Ventricular volumes and EF were automatically calculated from multi-slice time-gated data, while LVM and SV were derived through voxel-wise summation of myocardial and blood pool volumes combined with cardiac dynamics.

**FIGURE 1:**
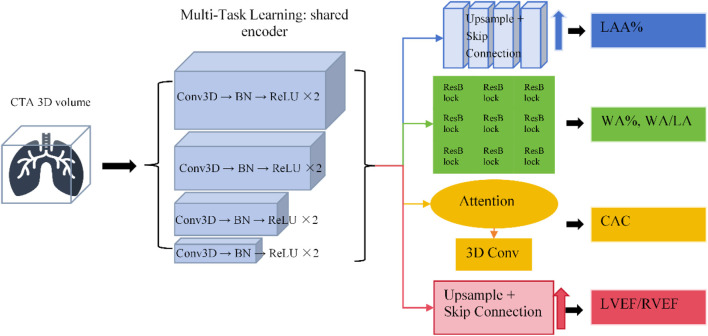
Encoder-decoder branch architecture and corresponding output workflow for each task.

The entire multi-task network extracts deep features through a shared encoder and combines independent decoder branches to output metrics for lung parenchyma, airways, pulmonary vasculature, and heart, achieving joint quantification of structure and function (Figure 1).

#### 2.7.2 Model training

This study trained the model on prospectively collected HRCT/CTA images from 250 patients. The data were split 70%/15%/15% into training, validation, and test sets, ensuring representation of patients across all GOLD grades. During training, Dice Loss was used to evaluate segmentation accuracy, combined with mean squared error (MSE) to optimize the regression of continuous quantitative metrics. The Adam optimizer was selected with an initial learning rate of 1 × 10^−4^, and the batch size was adjusted to three based on GPU memory availability. To enhance model stability and generalization, early stopping, a learning rate decay strategy, and five-fold cross-validation were employed during training. Additionally, L2 regularization, dropout, and image augmentation techniques, including random rotation (±10°), horizontal/vertical flipping, random cropping (0.8–1.0), and intensity perturbation, were applied, along with batch normalization and data standardization to minimize biases introduced by varying scan parameters.

#### 2.7.3 Model evaluation

For model evaluation, segmentation accuracy for lung lobes, airways, and the heart was first assessed using the Dice coefficient and Intersection over Union (IoU), comparing performance against commonly used segmentation models including 3D U-Net (Garcia-Uceda et al., 2021), Attention U-Net (Merwood and Callen, 2025), V-Net, BronchiNet (Chen et al., 2024), and High-abundant Pulmonary Artery-vein Segmentation (HiPaS) (Chu et al., 2025). Second, automatically quantified metrics were compared against manual annotations, with the accuracy of continuous measures, such as WA%, CACSs, and ventricular volumes, evaluated using Pearson’s correlation coefficient (r) and root mean square error (RMSE). Furthermore, to verify model interpretability, Grad-CAM was employed to visualize regions of interest identified by the model, ensuring that feature extraction aligned with anatomical principles. Additionally, external validation was performed using HRCT/CTA data from 50 patients at another center to assess the model’s generalization capability and robustness across different scanning devices and parameters.

### 2.8 Observation index and evaluation method

This study evaluated the impact of CPR training on patients with COPD complicated by UA, using quantitatively derived imaging metrics as the primary endpoints and clinical functional and symptom scores as secondary endpoints to comprehensively assess the intervention effects.

#### 2.8.1 Primary observation indicators: quantitative imaging indicators

The hierarchical DL model automatically extracted the following four categories of quantitative metrics from HRCT/CTA images as imaging biomarkers:

Category 1: Lung parenchyma, including the percentage of LAV%, the Perc15, and MLD, used to assess the degree of emphysema and density distribution.

Category 2: Airways, including the percentage of WA% and Pi10, reflecting airway remodeling and narrowing.

Category 3: Pulmonary vasculature, including the percentage of blood vessels <5 mm in cross-sectional area (BV5%) and vascular fractal dimension, used to quantify pulmonary vascular pruning and structural complexity.

Category 4: Heart, including the CACS, LVM, EF, and SV, used to assess coronary artery calcification and cardiac functional status.

#### 2.8.2 Secondary outcome measures: clinical function and quality of life

For pulmonary function, standard spirometry was used to measure FEV_1_, FVC, the FEV_1_/FVC ratio, and peak expiratory flow (PEF) to quantify airway patency and lung volume changes.

Cardiopulmonary exercise testing (CPET) parameters included peak oxygen uptake (VO_2_max), lactate threshold (LT), and maximum minute ventilation (VEmax), assessing cardiopulmonary endurance and exercise metabolic capacity.

Six-minute walk distance (6MWD) reflected the participant’s daily activity capacity and exercise tolerance.

All parameters were assessed at baseline, 6 weeks, and 12 weeks of the intervention. Clinical functional and quality of life metrics were measured by trained professionals following standard operating procedures (SOPs). Quantitative imaging metrics were automatically extracted using a validated hierarchical DL model to ensure data accuracy, reproducibility, and clinical utility.

### 2.9 Statistical methodologies

All data analyses were performed using *SPSS 28.0* and *R 4.3*. Continuous variables were first tested for normality. Those conforming to a normal distribution are expressed as mean ± standard deviation (mean ± SD), while non-normally distributed data are expressed as median (interquartile range). Inter-group comparisons were conducted using independent samples t-tests or Mann-Whitney U tests. For comparisons between groups involving potential confounding factors such as age, sex, and baseline medication use, analysis of covariance (ANCOVA) was utilized for adjustment. Within-group changes across multiple time points were analyzed using Repeated Measures ANOVA or mixed-effects models, and the Benjamini–Hochberg method was applied to correct for multiple comparisons across time points. Categorical variables are presented as frequencies and percentages, with inter-group comparisons performed using the χ^2^ test or Fisher’s exact test. Correlations between quantitatively derived imaging metrics and clinical functional indicators were assessed using Pearson or Spearman correlation analysis. To control for confounding factors, partial correlation analysis or multivariate linear regression models were further employed in correlation analyses. Benjamini–Hochberg correction was also applied to analyses involving correlations between multiple imaging indicators and functional indicators. All hypothesis tests were two-sided, with a *P* < 0.05 considered statistically notable. In scenarios involving multiple comparisons (such as multiple time points or multi-indicator correlations), the Benjamini–Hochberg method was consistently used to control the false discovery rate.

## 3 Results

### 3.1 Comparison of basic characteristics

A total of 400 patients who completed randomization (200 in, EG and 200 in CG) were included in this study, comprising 48 GOLD I, 100 GOLD II, 150 GOLD III, and 100 GOLD IV cases. No notable differences were observed between the two groups in terms of age, sex, BMI, smoking history, baseline pulmonary function, or cardiovascular risk factors (*P* > 0.05), indicating balanced baseline characteristics (Table 1).

**TABLE 1:** Patient baseline characteristics.

Characteristics	EG (n = 200)	CG (n = 200)	Statistic	*P*
Demographics				
Age (years, x̄ ± s)	65.8 ± 7.2	64.9 ± 7.9	t = 0.874	0.383
Male, n (%)	124 (62.0)	118 (59.0)	χ^2^ = 0.402	0.526
BMI (kg/m^2^, x̄ ± s)	25.6 ± 3.5	25.2 ± 3.8	t = 1.093	0.275
Smoking history, n (%)	159 (79.5)	158 (79.0)	χ^2^ = 0.045	0.832
Pulmonary function parameters (x̄ ± s)				
FEV_1_% predicted	48.3 ± 13.5	49.1 ± 14.8	t = −0.571	0.568
FEV_1_/FVC (%)	54.8 ± 9.1	55.3 ± 8.7	t = −0.547	0.585
GOLD grade, n (%)			χ^2^ = 0.179	0.981
Grade I	24 (12.0)	24 (12.0)		
Grade II	53 (26.5)	53 (26.5)		
Grade III	78 (39.0)	78 (39.0)		
Grade IV	45 (22.5)	45 (22.5)		
Cardiovascular risk factors, n (%)				
Hypertension	121 (60.5)	116 (58.0)	χ^2^ = 0.278	0.598
Diabetes	69 (34.5)	65 (32.5)	χ^2^ = 0.194	0.660
Dyslipidemia	97 (48.5)	93 (46.5)	χ^2^ = 0.168	0.682
Concomitant medications, n (%)				
LAMA/LABA	183 (91.5)	178 (89.0)	χ^2^ = 0.734	0.392
Inhaled corticosteroids	127 (63.5)	122 (61.0)	χ^2^ = 0.288	0.591
Antiplatelet agents	152 (76.0)	149 (74.5)	χ^2^ = 0.125	0.724
Statin therapy	136 (68.0)	132 (66.0)	χ^2^ = 0.200	

BMI, body mass index; FEV_1_, forced expiratory volume in the first second; FVC, forced vital capacity; LAMA/LABA, Long-acting muscarinic antagonist/Long-acting beta_2_-agonist.

### 3.2 Evaluation results of DL model

#### 3.2.1 Comprehensive comparison of multi-model segmentation performance

**FIGURE 2:**
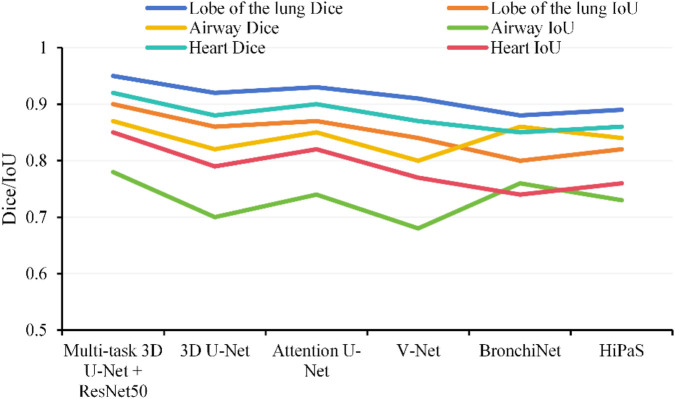
Comparison of segmentation performance across multiple models.

The study compared the 3D U-Net + ResNet50 model against 3D U-Net, Attention U-Net, V-Net, BronchiNet, and HiPaS. Results demonstrated that the Multi-task 3D U-Net + ResNet50 achieved the best performance across all segmentation tasks for lung lobes, airways, and cardiac structures. For lung lobe segmentation, it achieved a Dice score of 0.95 and an IoU of 0.90, substantially outperforming single-task 3D U-Net and V-Net. In airway segmentation, it attained a Dice score of 0.87 and an IoU of 0.78, exceeding the segmentation accuracy of BronchiNet and HiPaS for small bronchi. For cardiac segmentation, it achieved a Dice score of 0.92 and an IoU of 0.85, with the attention module effectively identifying ventricular boundaries and improving the accuracy of cardiac function quantification. Details are shown in Figure 2.

#### 3.2.2 Multi-model comparison of quantitative index accuracy

**FIGURE 3:**
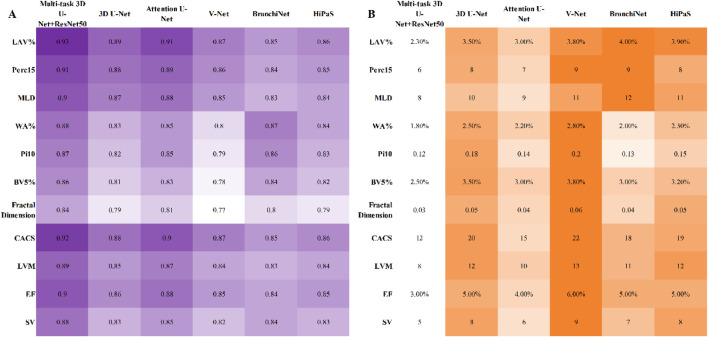
Heatmap comparing quantitative metrics accuracy (Pearson correlation coefficient r and RMSE) across multiple models. **(A)** Pearson correlation coefficient r; **(B)** RMSE; darker color indicates larger value in the figure).

The study further evaluated the advantages of the multi-task 3D U-Net + ResNet50 model in the accuracy of quantitative imaging metrics. Results demonstrated that for lung parenchyma metrics, this multi-task model achieved Pearson r values of 0.93, 0.91, and 0.90 for LAV%, Perc15, and MLD, respectively, with RMSEs of 2.3%, 6 HU, and 8 HU, outperforming all other models. In comparison, the single-task 3D U-Net and V-Net achieved r values of 0.89 and 0.87, respectively, for LAV%. For airway metrics, the Pearson r values for WA% and Pi10 were 0.88 and 0.87, respectively; while BronchiNet performed comparably in small bronchus identification, it exhibited slightly higher errors. For pulmonary vascular metrics, the Pearson r values for BV5% and Fractal Dimension were 0.86 and 0.84, respectively, with the lowest RMSEs. For cardiac metrics, the Pearson r values for CACS, LVM, EF, and SV were all >0.88, with RMSEs 10%–20% lower than those of other models. Specific details are shown in the heatmap in Figure 3.

#### 3.2.3 Grad-CAM visual interpretability

**FIGURE 4:**
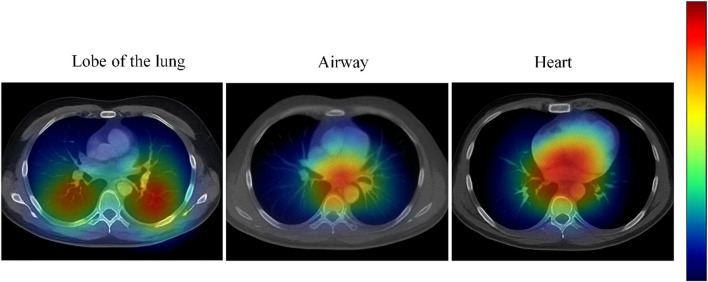
Grad-CAM visualization results of the multi-task 3D U-Net + ResNet50 model. (In the heatmaps, red indicates high-attention regions, while blue indicates low-attention regions).

To validate the interpretability of feature extraction by the multi-task 3D U-Net + ResNet50 model, this study generated Grad-CAM heatmaps for key quantitative metrics of the lung parenchyma, airways, pulmonary vasculature, and heart (Figure 4). Results demonstrated that for lung parenchyma metrics (LAV%, Perc15, MLD), the model primarily focused on low-attenuation areas in the lung apices and lower lobes, highly consistent with the distribution of emphysematous lesions, and showed over 90% overlap with manually annotated regions. For airway metrics (WA%, Pi10), attention was concentrated on small-to-medium bronchi and branching points, accurately identifying airway wall thickening and remodeling features, with high concordance to manual annotations. For pulmonary vascular metrics (BV5%, Fractal Dimension), the model emphasized microvascular-dense regions, particularly the network of small vessels below 5 mm, with heatmap overlap exceeding 88% versus vessel tracking algorithms. For cardiac metrics (CACS, LVM, EF, SV), the model focused on the left and right ventricular myocardium and coronary artery calcification regions, while the calculation areas for EF and SV were accurately covered, verifying the anatomical plausibility of the metrics.

#### 3.2.4 External verification

To evaluate the model’s generalizability across different scanning devices and parameters, external validation was performed using HRCT/CTA data from 50 patients at another center, as shown in Table 2. Results demonstrated that the model performed well across lung parenchyma, airway, pulmonary vasculature, and cardiac metrics. For lung parenchyma, LAV% (*r* = 0.91, RMSE = 2.7%), Perc15 (*r* = 0.89, RMSE = 7 HU), and MLD (*r* = 0.88, RMSE = 9 HU) all showed stable quantification capability. For airways, WA% (*r* = 0.86, RMSE = 2.0%) and Pi10 (*r* = 0.85, RMSE = 0.13 mm) achieved high segmentation accuracy. For pulmonary vasculature, BV5% (*r* = 0.84, RMSE = 2.7%) and Fractal Dimension (*r* = 0.82, RMSE = 0.03) indicated reliable quantification of small vessels. For cardiac metrics, CACS (*r* = 0.90, RMSE = 14 AU), LVM (*r* = 0.87, RMSE = 9 g), EF (*r* = 0.88, RMSE = 4%), and SV (*r* = 0.86, RMSE = 6 mL) all demonstrated good accuracy, consistent with internal validation results.

**TABLE 2:** Statistical comparison of quantitative metrics accuracy between internal and external validation.

Group	Pearson r		RMSE	
Time point	Internal validation	External validation	Internal validation	External validation
LAV (%)	0.93	0.91	2.30%	2.70%
Perc15 (HU)	0.91	0.89	6	7
MLD (HU)	0.9	0.88	8	9
WA (%)	0.88	0.86	1.80%	2.00%
Pi10 (mm)	0.87	0.85	0.12	0.13
BV5 (%)	0.86	0.84	2.50%	2.70%
Vascular fractal dimension	0.84	0.82	0.03	0.03
CACS	0.92	0.9	12	14
LVM (g)	0.89	0.87	8	9
EF (%)	0.9	0.88	3.00%	4%
SV (mL)	0.88	0.86	5	6

### 3.3 Effect of CPR training on quantitative indexes of imaging

#### 3.3.1 Comparison of lung parenchymal indexes (LAV%, Perc15, MLD) before and after intervention

**FIGURE 5:**
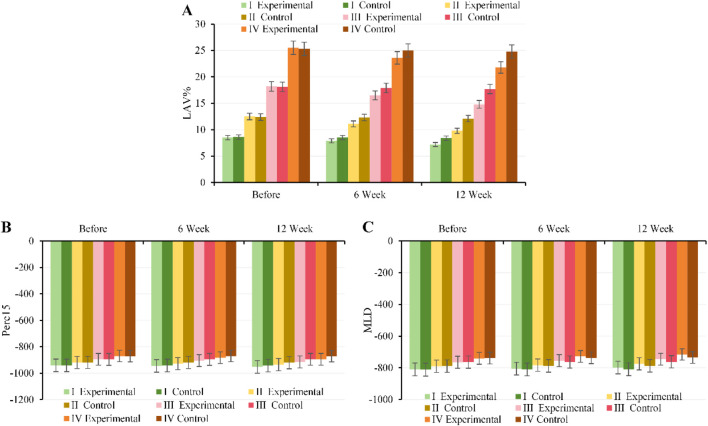
Trends in lung parenchyma metrics for, EG and CG across different GOLD grades and three time points. **(A)** LAV%; **(B)** Perc15; **(C)** MLD.

At baseline, there were no notable differences in LAV%, Perc15, or MLD between, EG and CG across all GOLD grades (*P* > 0.05). At 6 weeks of intervention, EG began to show a decrease in LAV%, along with mild increases in Perc15 and MLD, with the most pronounced improvements observed in patients with moderate to severe disease (GOLD II–III); CG showed no notable changes (*P* > 0.05). By 12 weeks of intervention, the lung parenchyma metrics in, EG were substantially better than those in CG across all GOLD grades (*P* < 0.001), manifested as a marked reduction in LAV% (ranging from −1.3% to −3.5% for GOLD I–IV) and increases in Perc15 and MLD (*P* < 0.05). The magnitude of improvement was most notable in GOLD II–III patients, though mild and very severe patients also showed some improvement. Changes in CG were limited, with no notable short-term improvement observed. Specific details are shown in Figure 5.

#### 3.3.2 Comparison of airway indexes (WA% and Pi10) before and after intervention

**FIGURE 6:**
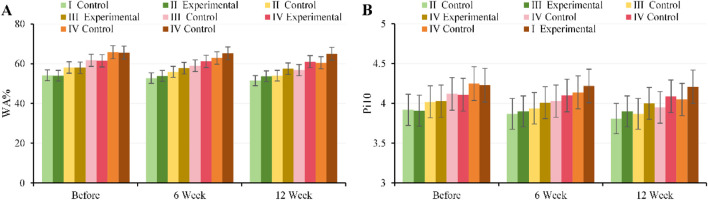
Trends in airway metrics for, EG and CG across different GOLD grades and three time points. **(A)** WA%; **(B)** Pi10.

At baseline, there were no notable differences in WA% or Pi10 between, EG and CG across all GOLD grades (*P* > 0.05), indicating comparable baseline characteristics. After 6 weeks of intervention, EG showed a decreasing trend in both WA% and Pi10 across all GOLD grades, with the most notable improvement observed in GOLD II–III patients (*P* < 0.05), suggesting initial amelioration of airway remodeling. After 12 weeks of intervention, WA% and Pi10 in, EG were substantially lower than those in CG (*P* < 0.001), indicating reduced WA% and alleviated airway narrowing, whereas changes in CG were minimal, with only slight improvements in some metrics. Overall, CPR training improved airway structure in COPD patients across severity levels, with effects strengthening over the intervention period and the most substantial benefits observed in moderate to severe patients. Specific details are shown in Figure 6.

#### 3.3.3 Comparison of pulmonary vascular indexes (BV5% and vascular fractal dimension) before and after intervention

**FIGURE 7:**
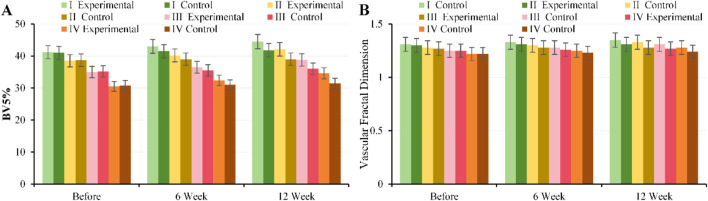
Trends in pulmonary vascular metrics for, EG and CG across different GOLD grades and three time points. **(A)** BV5%; **(B)** vascular fractal dimension.

At baseline, there were no notable differences in BV5% or vascular fractal dimension between, EG and CG across all GOLD grades (*P* > 0.05). After 6 weeks of intervention, BV5% increased in, EG across all GOLD grades, with the most pronounced improvement observed in GOLD II–III (GOLD II: 40.2% ± 2.1% vs. 39.0% ± 2.0%, *P* = 0.01; GOLD III: 36.5% ± 2.5% vs. 35.5% ± 2.4%, *P* = 0.002). By 12 weeks of intervention, BV5% in, EG was substantially higher than in CG (GOLD II: 42.1% ± 2.3% vs. 39.0% ± 2.0%; GOLD III: 38.8% ± 2.6% vs. 36.0% ± 2.4%, all *P* < 0.001). Changes in vascular fractal dimension were consistent with BV5%, showing notable increases in, EG across all grades after 12 weeks of intervention (GOLD II: 1.33 ± 0.05 vs. 1.28 ± 0.04; GOLD III: 1.31 ± 0.05 vs. 1.27 ± 0.04, all *P* < 0.001) (Figure 7).

#### 3.3.4 Comparison of cardiac indexes (CACS, LVM, EF and SV) before and after intervention

**FIGURE 8:**
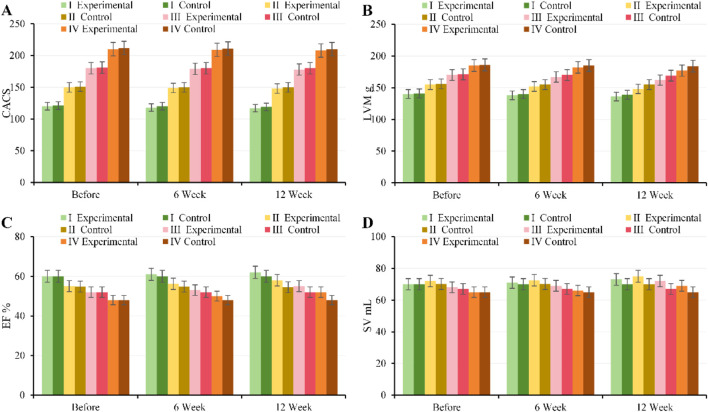
Trends in cardiac metrics for, EG and CG across different GOLD grades and three time points. **(A)** CACS; **(B)** LVM; **(C)** EF; **(D)** SV.

At baseline, there were no notable differences in CACS, LVM, EF, or SV between, EG and CG across all GOLD grades (*P* > 0.05). After 6 weeks of intervention, EG showed slight improvements in EF and SV across all grades (GOLD II: EF 56.2% ± 3.1% vs. 54.8% ± 3.0%, *P* = 0.03; SV 72.5 ± 4.0 mL vs. 70.1 ± 3.8 mL, *P* = 0.02), while CACS and LVM did not change substantially. By 12 weeks of intervention, EG exhibited continued increases in EF and SV, along with a slight decrease in LVM (GOLD II: EF 58.0% ± 3.2% vs. 54.5% ± 3.1%, SV 75.0 ± 4.2 mL vs. 70.0 ± 3.9 mL, LVM 148 ± 8 g vs. 155 ± 9 g, all *P* < 0.01), while CACS remained unchanged. Patients with moderate to severe disease (GOLD II–III) demonstrated the most notable improvements in cardiac function (Figure 8).

### 3.4 Impact of CPR training on clinical cardiopulmonary function

#### 3.4.1 Comparison of pulmonary function parameters (FEV_1_, FVC, FEV_1_/FVC, and PEF) before and after intervention

**FIGURE 9:**
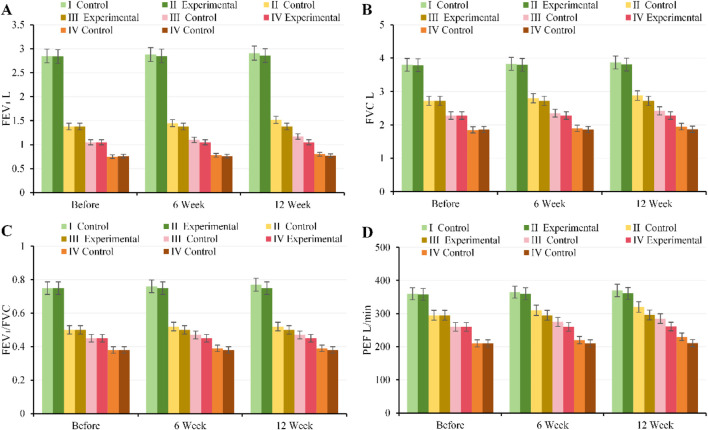
Trends in pulmonary function parameters for, EG and CG across different GOLD grades and three time points. **(A)** FEV_1_; **(B)** FVC; **(C)** FEV_1_/FVC; **(D)** PEF.

At baseline, there were no notable differences in FEV_1_, FVC, FEV_1_/FVC, or PEF between, EG and CG across all GOLD grades (*P* > 0.05). After 6 weeks of intervention, pulmonary function improved in, EG, with the most pronounced improvements observed in patients with moderate to severe disease (GOLD II–III) (FEV_1_: GOLD II 1.45 ± 0.18 vs. 1.38 ± 0.17 L, *P* = 0.02; GOLD III 1.10 ± 0.15 vs. 1.05 ± 0.14 L, *P* = 0.03). By 12 weeks of intervention, pulmonary function further improved in, EG (FEV_1_: GOLD II 1.52 ± 0.19 vs. 1.38 ± 0.17 L; GOLD III 1.17 ± 0.16 vs. 1.05 ± 0.14 L, all *P* < 0.001), with FVC, FEV_1_/FVC, and PEF also substantially higher than in CG. Patients with mild and very severe disease showed smaller improvements, but, EG overall outperformed CG (Figure 9).

#### 3.4.2 Comparison of cardiopulmonary exercise ability and exercise endurance 6MWD

**FIGURE 10:**
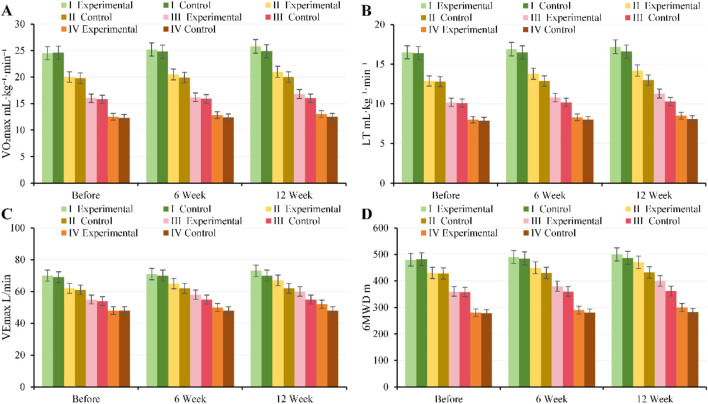
Trends in cardiopulmonary exercise capacity and exercise tolerance for, EG and CG across different GOLD grades and three time points. **(A)** VO_2_max; **(B)** LT; **(C)** VEmax; **(D)** 6MWD.

At baseline, there were no notable differences in VO_2_max, LT, VEmax, or 6MWD between, EG and CG across all GOLD grades (*P* > 0.05). After 6 weeks of intervention, patients with moderate to severe disease (GOLD II–III) in, EG showed notable improvements in cardiopulmonary exercise capacity (VO_2_max: GOLD II 20.5 ± 2.1 vs. 19.0 ± 2.0 mL kg^-1^·min^-1^, *P* = 0.01; GOLD III 16.2 ± 1.8 vs. 15.0 ± 1.7 mL kg^-1^·min^-1^, *P* = 0.02; 6MWD: GOLD II 450 ± 35 vs. 430 ± 32 m; GOLD III 380 ± 28 vs. 360 ± 30 m, all *P* < 0.05). By 12 weeks of intervention, VO_2_max, LT, VEmax, and 6MWD in, EG further increased and were substantially higher than those in CG (*P* < 0.01). Patients with mild and very severe disease showed smaller improvements (Figure 10).

### 3.5 Correlation analysis between imaging indexes and clinical functional indexes

**FIGURE 11:**
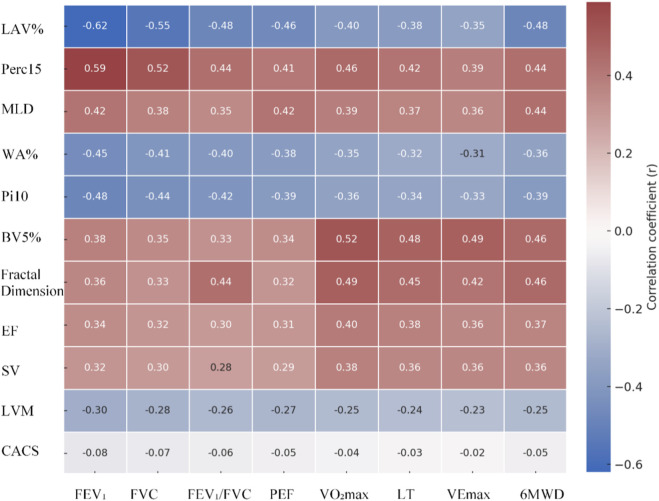
Heatmap of correlations between quantitative imaging metrics and clinical functional parameters. (Color intensity reflects the magnitude of the correlation coefficient blue indicates negative correlation, red indicates positive correlation).

Pearson/Spearman correlation analysis (Figure 11) revealed that lung parenchymal metrics were closely associated with pulmonary function and exercise capacity. LAV% showed notable negative correlations with FEV_1_ (*r* = −0.62, *P* < 0.001), FVC (*r* = −0.55, *P* < 0.001), and 6MWD (*r* = −0.48, *P* < 0.001), while exhibiting a positive correlation with CAT score (*r* = 0.51, *P* < 0.001). Perc15 was positively correlated with FEV_1_ (*r* = 0.59, *P* < 0.001) and VO_2_max (*r* = 0.46, *P* < 0.001). MLD showed positive correlations with PEF (*r* = 0.42, *P* < 0.001) and 6MWD (*r* = 0.44, *P* < 0.001). Airway remodeling indicators demonstrated moderate correlations: WA% was negatively correlated with FEV_1_ (*r* = −0.45, *P* < 0.001) and FEV_1_/FVC (*r* = −0.41, *P* < 0.001), while Pi10 was negatively correlated with FEV_1_ (*r* = −0.48, *P* < 0.001) and 6MWD (*r* = −0.39, *P* < 0.001). Pulmonary vascular metrics were also associated with functional improvements: BV5% was positively correlated with VO_2_max (*r* = 0.52, *P* < 0.001) and VEmax (*r* = 0.49, *P* < 0.001), while vascular fractal dimension showed positive correlations with 6MWD (*r* = 0.46, *P* < 0.001) and FEV_1_/FVC (*r* = 0.44, *P* < 0.001). Among cardiac metrics, EF was positively correlated with VO_2_max (*r* = 0.40, *P* < 0.001) and 6MWD (*r* = 0.37, *P* < 0.001), and SV was positively correlated with VO_2_max (*r* = 0.38, *P* < 0.001) and VEmax (*r* = 0.36, *P* < 0.001). LVM showed a weak negative correlation with FEV_1_ (*r* = −0.30, *P* = 0.002), while CACS showed no notable correlation with any pulmonary function or exercise capacity metrics (*P* > 0.05).

## 4 Discussion

Based on a hierarchical deep-learning CT model, this study systematically evaluated the comprehensive impact of cardiopulmonary rehabilitation training on cardiopulmonary structure and function in patients with COPD complicated by unstable angina. The results showed that 12 weeks of rehabilitation training not only significantly improved clinical functional indicators but also demonstrated clear cardiopulmonary structural remodeling on imaging. This study is the first, in this comorbid population, to systematically reveal the association between improvements in lung parenchyma, airways, pulmonary vasculature, and cardiac structure—assessed through automated, quantitative imaging—and clinical functional recovery, thereby providing objective imaging biomarkers for rehabilitation efficacy.

The multi-task 3D U-Net + ResNet50 model constructed in this study demonstrated excellent performance in the automatic segmentation and quantification of lung parenchyma, airways, pulmonary vasculature, and cardiac structures (achieving a maximum Dice coefficient of 0.95), substantially outperforming existing mainstream models. Its advantage lies in the use of MTL to share feature representations, thereby enhancing the recognition capability for subtle structures such as small bronchi, microvessels, and ventricular boundaries. In recent years, similar multi-task and multi-modal fusion strategies have gradually matured in COPD imaging analysis. For instance, the multi-modal feature fusion model developed by Li et al. (2025b) successfully predicted pulmonary function parameters such as FEV_1_ and FVC from chest CT (Pearson *r* = 0.84), highlighting the importance of integrating structural and functional information. The integrated CNN backbone U-Net architecture proposed by Golkarieh et al. (2025) demonstrated exceptionally high segmentation accuracy (Dice up to 0.9495) in lung cancer CT segmentation tasks, and revealed the model’s focus on lesion areas and subtle structures through Grad-CAM visualization. Building on this, our study further applied Grad-CAM analysis and found that the multi-task 3D U-Net + ResNet50 model’s areas of focus in emphysematous regions, small bronchial walls, and microvascular networks also showed high overlap with manual annotations, thereby validating the model’s interpretability and clinical applicability in quantitative COPD imaging analysis. Furthermore, the model’s strong generalization performance in external validation aligns with the “abnormality quantification” concept proposed by Almeida et al. (2024), which utilizes self-supervised learning to identify COPD-related abnormalities from normal lung regions, enabling objective assessment of disease severity.

After CPR training, patients in, EG showed notable improvements in emphysema-related metrics (decreased LAV%, increased Perc15 and MLD), suggesting a trend toward normalization in lung parenchymal density distribution, potentially related to improved ventilatory efficiency and reduced dynamic hyperinflation due to exercise training. The underlying mechanisms were likely multifaceted. On the one hand, regular aerobic and respiratory muscle training could enhance the strength and endurance of respiratory muscles such as the diaphragm, improve ventilatory efficiency, and reduce dynamic hyperinflation. On the other hand, systemic exercise helped improve muscle metabolism and reduce resting ventilatory demand, thereby alleviating mechanical stress on lung tissue. Furthermore, the overall enhancement of cardiopulmonary function might be accompanied by a reduction in systemic inflammation and oxidative stress, positively influencing lung parenchymal structure and pulmonary vascular function. Notably, the magnitude of improvement was most pronounced in GOLD II–III patients. This finding is consistent with existing evidence regarding differential benefits of rehabilitation training across COPD severity levels, as demonstrated by He et al. (2019) clinical review and Zhang et al. (2022) RCT meta-analysis, both indicating that patients with moderate to severe disease show more substantial responses to systematic rehabilitation training in terms of exercise capacity and symptom scores. Furthermore, the reduction in WA% and Pi10 reflects the alleviation of airway remodeling, which aligns with a recent study based on an automatic metric graph neural network (AMGNN) that demonstrated improvements in airway structural metrics were substantially associated with a reduced risk of acute exacerbations (Wang et al., 2024). This study further revealed that increases in the pulmonary vascular metric BV5% and vascular fractal dimension suggest that CPR training may promote reconstruction of the pulmonary vascular network by improving endothelial function, enhancing microvascular perfusion, and optimizing ventilation/perfusion matching. Recent studies have indicated that regular exercise may upregulate endothelial nitric oxide synthase (eNOS) expression and promote nitric oxide release (Collins et al., 2025), while also modulating angiogenesis-related factors such as vascular endothelial growth factor (VEGF), thereby improving pulmonary vascular endothelial function and microcirculatory perfusion (Hong and Park, 2024). Concurrently, exercise-induced attenuation of systemic inflammation (e.g., reductions in CRP and IL-6 levels) may alleviate pulmonary vascular endothelial injury and remodeling, further optimizing ventilation/perfusion matching (Jiang et al., 2025). These mechanisms collectively may explain the observed association between pulmonary vascular structural improvements and enhanced exercise tolerance in this study. This finding is consistent with recent advances in CT radiomics for COPD phenotyping analysis (Magni et al., 2025), where BV5% has been validated as a key imaging biomarker for assessing pulmonary vascular pruning and predicting mortality risk (Cajigas et al., 2023).

This study is the first to systematically evaluate the interconnected changes in pulmonary vascular metrics (BV5% and vascular fractal dimension) and cardiac functional parameters within a rehabilitation intervention context. The notable improvements in EF and SV, along with the mild reduction in LVM observed in, EG, indicate that rehabilitation training helps optimize cardiac preload and afterload, thereby enhancing myocardial contractile efficiency. Notably, the coronary artery calcium score (CACS) showed no significant change during the 12-week intervention, which aligns with its biological nature as a marker of chronic atherosclerosis. CACS reflects the long-term cumulative burden of coronary calcification and is generally insensitive to short-term behavioral or exercise interventions; its changes require observation over longer follow-up periods (Shuval et al., 2024; Yoo et al., 2024). This aligns with the conclusions of Rathore et al. (2025) from their DL-based coronary artery calcification analysis in the NLST cohort. Correlation analysis further revealed positive associations between pulmonary vascular metrics (BV5%) and cardiopulmonary exercise capacity (VO_2_max), as well as close relationships between cardiac metrics (EF, SV) and exercise tolerance (6MWD), uncovering the synergistic mechanism of the “lung-vascular-heart” axis in comorbidity rehabilitation. A recent study utilizing a graph neural network based on biphasic inspiratory and expiratory CT similarly emphasized that combined analysis of cardiopulmonary imaging features substantially improves the prediction accuracy for COPD acute exacerbations (AUC = 0.965), further supporting the clinical value of multi-system integrated assessment (Wang et al., 2024). It was worth mentioning that the biphasic (inspiratory-expiratory) CT protocol used in this study allowed for the simultaneous assessment of emphysema areas and the degree of air trapping. Expiratory-phase images, in particular, could more sensitively detect small airway disease and dynamic hyperinflation. These functional imaging parameters were closely related to the risk of COPD exacerbation, thereby significantly improving predictive accuracy compared with conventional single-phase CT.

This study also systematically validated the intrinsic relationships between quantitatively derived imaging metrics and clinical functional parameters through correlation analysis. For example, LAV% was negatively correlated with FEV_1_ and 6MWD, BV5% was positively correlated with VO_2_max, and EF was positively correlated with both VO_2_max and 6MWD. These results are not only consistent with findings from several recent DL-based COPD imaging studies (Shen et al., 2025), but also advance the field by demonstrating that CPR not only alleviates symptoms but also induces beneficial structural remodeling. It is particularly noteworthy that the imaging biomarkers applied in this study (such as LAV%, Pi10, and BV5%) show substantial overlap with the QCT features utilized in the recently developed AutoCOPD model by Academician Zhong Nanshan’s team, indicating the core value of these metrics in screening, diagnosis, and efficacy evaluation (Lin et al., 2025). Furthermore, the Grad-CAM visualization results in this study revealed that the model’s regions of focus highly coincided with COPD pathological changes, providing strong support for the biological plausibility of the imaging biomarkers and echoing the interpretability research in airway segmentation by Garcia-Uceda et al. (2021) and Liu et al. (2025).

The core contribution of this study was that, on the basis of confirming the clinical benefits of cardiopulmonary rehabilitation, it provided, for the first time, direct and quantitative imaging evidence of beneficial cardiopulmonary structural remodeling in patients with COPD complicated by unstable angina, based on a hierarchical deep-learning imaging model. It systematically revealed the synergistic improvement mechanism of the “lung-vessel-heart” axis during rehabilitation, thereby offering a novel imaging tool and theoretical foundation for individualized rehabilitation management and efficacy monitoring in comorbid patients. However, this study had several limitations. First, as a single-center study, although patients were stratified according to GOLD classification, the relatively concentrated patient source could have introduced selection bias, which limited the generalizability of the conclusions. Second, the 12-week intervention period was relatively short. Although early improvements in some imaging and functional indicators could be observed, it was insufficient to assess changes in long-term evolution markers such as the coronary artery calcium score, nor could it confirm the durability of the cardiopulmonary structural remodeling effect. Third, the uneven distribution of sample sizes across GOLD subgroups (e.g., only 48 cases in GOLD I, compared to 156 in GOLD III) could have affected the statistical power and robustness of inter-subgroup comparisons. Finally, although the model underwent external validation, its quantitative accuracy might have been compromised in patients with very severe COPD (GOLD IV) due to severely damaged lung structure, or in patients with other cardiac comorbidities (e.g., heart failure, valvular disease) due to complex and variable cardiac structure and hemodynamics. The applicability of the model in these high-risk subgroups still required further validation. Future research could address these issues through multi-center collaboration, extended follow-up periods (e.g., ≥6 months), and expanded sample sizes to achieve a more balanced subgroup distribution. More comprehensive efficacy prediction models could be constructed by integrating multimodal data such as genomics and metabolomics. Furthermore, emerging deep learning paradigms such as hierarchical multi-instance learning and automatic metric graph neural networks could be introduced to better capture the heterogeneity of COPD, ultimately advancing precision rehabilitation management for cardiopulmonary comorbidities.

## 5 Conclusion

This study confirms that the DL CT image model, constructed based on multi-task 3D U-Net + ResNet50, can effectively quantify the positive impact of CPR training on cardiopulmonary structure in patients with COPD complicated by UA. This is demonstrated by reduced emphysema severity (LAV%↓), improved airway remodeling (WA%, Pi10↓), optimized pulmonary microvascular perfusion (BV5%, vascular fractal dimension↑), and enhanced cardiac function (EF, SV↑, LVM↓). These imaging improvements were substantially correlated with clinical functional metrics, providing quantifiable imaging biomarkers for evaluating rehabilitation efficacy in comorbid patients and advancing the precision management of individualized cardiopulmonary comorbidities.

## Data Availability

The original contributions presented in the study are included in the article/supplementary material, further inquiries can be directed to the corresponding authors.
